# Morphological and molecular diversity in mid-late and late maturity genotypes of cauliflower

**DOI:** 10.1371/journal.pone.0290495

**Published:** 2023-08-31

**Authors:** Neha Rana, Akhilesh Sharma, Ranbir Singh Rana, Hem Lata, Bansuli, Alisha Thakur, Vivek Singh, Aditya Sood

**Affiliations:** 1 Department of Vegetable Science & Floriculture, Chaudhary Sarwan Kumar Himachal Pradesh Agricultural University, Palampur, Himachal Pradesh, India; 2 Centre for Geo Informatics Research and Training, Chaudhary Sarwan Kumar Himachal Pradesh Krishi Vishvavidyalaya, Palampur, Himachal Pradesh, India; ICAR Research Complex for North Eastern Hill Region Manipur Centre, INDIA

## Abstract

Genetic diversity is the prerequisite for the success of crop improvement programmes. Keeping in view, the current investigation was undertaken to assess the agro-morphological and molecular diversity involving 36 diverse mid-late and late cauliflower genotypes following α-RBD design during winter season 2021–22. Six morphological descriptors predicted as polymorphic using Shannon diversity index with maximum for leaf margin (0.94). The genotypes grouped into nine clusters based on D^2^ analysis with four as monogenotypic and gross plant weight (32.38%) revealed maximum contribution towards the genetic diversity. Molecular diversity analysis revealed 2–7 alleles among 36 polymorphic simple sequence repeats (SSR) with average of 4.22. Primer BoESSR492 (0.77) showed maximum polymorphic information content (PIC) with mean of 0.58. SSR analysis revealed two clusters each with two subclusters with a composite pattern of genotype distribution. STRUCTURE analysis showed homogenous mixture with least amount of gene pool introgression within the genotypes. Thus, based on morphological and molecular studies, the diverse genotypes namely, DPCaCMS-1, DPCaf-W4, DPCaf-US, DPCaf-W131W, DPCaf-S121, DPCaf-18, DPCaf-13, DPCaf-29 and DPCaf-CMS5 can be utilized in hybridization to isolate potential transgressive segregants to broaden the genetic base of cauliflower or involve them to exploit heterosis.

## Introduction

Amongst the cole crops belonging to family Brassicaceae, cauliflower (*Brassica oleracea* var. *botrytis* L., 2n = 2x = 18) finds most significant place having low calories and high in dietary fibre, with a widespread center of origin in the Mediterranean Basin [1] and is grown in several nations across the world for both its nutritional content and for its added-value foods in the processing industry. Cauliflower is cultivated to obtain snow-white, compact and tender curds that are high in flavonoids, isothiocyanates, carotenoids, indoles and tocopherols which are frequently used as a vegetable in soups, curries and pickles [2]. Additionally, cauliflower has a variety of therapeutic uses, such as anti-cancer [3], lowering the risk of cardiovascular disease, and treating diabetes [4]. With an area, production, and productivity of 467 thousand hectares, 8941 thousand metric tonnes and 19.14 metric tonnes per hectare, respectively, India is the second-largest producer of cauliflower in the world after China [5].

Breeders have focused a lot of attention on the crop for varietal/hybrid development since it is a cost-effective, nutrient-dense vegetable crop with a wide range of plant and curd traits [6] and the variability in the germplasm is prerequisite to meet these objectives [7]. Thus, understanding the variable characteristics of the germplasm and their genetic relationships is crucial in crop development efforts to create new varieties, and enhancement of productivity [8]. This will make it easier to tackle upcoming difficulties including climate change, the depletion of natural resources and numerous biotic and abiotic stresses [9].

The Indian cauliflower germplasm is greatly diversified by the involvement of exotic germplasm and locally generated inter-group progenies for its utilization in heterosis breeding and stress resistance [10]. The present day cultivated cauliflower varieties in India is the result of unique genomic composition of the Indian cauliflower and snowball types. The late cauliflower in India is the consequence of introduction from European countries and have played a significant role in the evolution of mid-late group that shares resemblance of both snowball and typical Indian types [11]. The introgression of genes (major/minor) during the isolation of new genotypes alter the performance of different attributes for their adaptability and consumer acceptance. The researchers reported wide diversity in various maturity groups of cauliflower viz., early [12], mid group [13], and snowball type [14–16] using morphological descriptors. However, the traits across groups are sensitive to environmental conditions and thereby, these observations do not satisfactorily reproduce degree of variation in the genotypes for use in breeding. The understanding of degree of variation at molecular level and corroborating this knowledge with morphological descriptors would provide enormous significance to the breeders in planning their breeding program. The plant architecture of cauliflower germplasm exhibited enormous diversity for leaf and curd characteristics in terms of size, shape, colour and compactness [17] that would be available using different methodologies i.e. evaluations of morphological features, isozymes, total seed protein and molecular markers [1]. The assessment of genetic diversity and relationships of germplasm through morphological characterization is the first step in any breeding program [18], although they involve lots of labour and time besides sensitive to environmental factors [17]. Molecular markers are therefore viewed as the best tools to understand genetic relationships in crop species as they are independent of environmental variables, dominant/co-dominant in nature and suggest precise scoring techniques [18]. Microsatellite markers are the best in comparison to other markers on account of easy detection through PCR, ample number, highly polymorphic, co-dominantly inherited, multi-allelic and consistently dispersed in genomes and require small amount of DNA for analysis [19]. In order to estimate the genetic diversity in cauliflower germplasm, it would be of immense significance to understand the variation at the molecular level and their validation using morphological descriptors. Keeping in view the above aspects, the current study was planned with objectives to assess morphological variation and molecular diversity using SSR markers in genotypes of mid-late and late cauliflower.

## Material and methods

### Plant materials

#### Experimental layout

Thirty-six mid-late and late cauliflower genotypes were used to study genetic diversity and relationship determination derived from diverse populations and are also collected from diverse ecological areas (Table 1). These genotypes were assessed during winter of 2021–2022 at Vegetable Research Farm, Department of Vegetable Science and Floriculture, CSK Himachal Pradesh Agricultural University, Palampur using α–lattice square design using three replications, nine blocks per replication, and four entries per block. The pH of the soil in the experimental field was 5.7, making it an acidic clay loam. The seedlings were transplanted at 45 cm each as inter- and intra-row spacing. The seedling of 36 genotypes were raised in nursery beds of size 3 m × 1 m × 0.15 m on September 3, 2021 and were ready for transplanting in about 40 days.

**Table 1:** List of genotypes along with their source.

Sr. No.	Genotype	Pedigree	Source
1.	**DPCaf-US**	Selection from segregating open pollinated population of unknown hybrid	CSK Himachal Pradesh Krishi Vishvavidyalaya, Palampur
2.	**DPCaf-S121W**	Selection from segregating population of Snow Crown	CSK Himachal Pradesh Krishi Vishvavidyalaya, Palampur
3.	**DPCaf-S121**	Selection from segregating population of Snow Crown	CSK Himachal Pradesh Krishi Vishvavidyalaya, Palampur
4.	**DPCaf- S122**	Selection from segregating population of Snow Crown	CSK Himachal Pradesh Krishi Vishvavidyalaya, Palampur
5.	**DPCaf-S5-1**	Selection from segregating population of Snow Crown	CSK Himachal Pradesh Krishi Vishvavidyalaya, Palampur
6.	**DPCaf-W131W**	Selection from segregating population of White Excel	CSK Himachal Pradesh Krishi Vishvavidyalaya, Palampur
7.	**DPCaf-W4**	Selection from segregating population of White Excel	CSK Himachal Pradesh Krishi Vishvavidyalaya, Palampur
8.	**DPCaY-1**	Selection from Palam Uphar × Pusa Snowball K-1	CSK Himachal Pradesh Krishi Vishvavidyalaya, Palampur
9.	**DPCaY-4**	Selection from Pusa Sharad × Pusa Snowball K-1	CSK Himachal Pradesh Krishi Vishvavidyalaya, Palampur
10.	**DPCaY-7**	Selection from Pusa Sharad × Pusa Snowball K-1	CSK Himachal Pradesh Krishi Vishvavidyalaya, Palampur
11.	**DPCaY-9**	Selection from Palam Uphar × Pusa Snowball K-1	CSK Himachal Pradesh Krishi Vishvavidyalaya, Palampur
12.	**DPCaf-1**	Selection from natural open pollination population of different inbred	CSK Himachal Pradesh Krishi Vishvavidyalaya, Palampur
13.	**DPCaf-2**	Selection from natural open pollination population of different inbred	CSK Himachal Pradesh Krishi Vishvavidyalaya, Palampur
14.	**DPCaf-8**	Selection from Pusa Himjyoti × Pusa Snowball K-1	CSK Himachal Pradesh Krishi Vishvavidyalaya, Palampur
15.	**DPCaf-9**	Selection from natural open pollination population of different inbred	CSK Himachal Pradesh Krishi Vishvavidyalaya, Palampur
16.	**DPCaf-10**	Selection from Pusa Sharad × Palam Uphar	CSK Himachal Pradesh Krishi Vishvavidyalaya, Palampur
17.	**DPCaf-12**	Selection from natural open pollination population of different inbred	CSK Himachal Pradesh Krishi Vishvavidyalaya, Palampur
18.	**DPCaf-12-1**	Selection from natural open pollination population of different inbred	CSK Himachal Pradesh Krishi Vishvavidyalaya, Palampur
19.	**DPCaf-13**	Selection from Pusa Deepali × Palam Uphar	CSK Himachal Pradesh Krishi Vishvavidyalaya, Palampur
20.	**DPCaf-18**	Selection from natural open pollination population of different inbred	CSK Himachal Pradesh Krishi Vishvavidyalaya, Palampur
21.	**DPCaf-29**	Selection from Pusa Sharad × Pusa Snowball K-1	CSK Himachal Pradesh Krishi Vishvavidyalaya, Palampur
22.	**DPCaf-30**	Selection from natural open pollination population of different inbred	CSK Himachal Pradesh Krishi Vishvavidyalaya, Palampur
23.	**DPCaCMS-1**	Derived through back cross breeding of CMS line × Palam Uphar	CSK Himachal Pradesh Krishi Vishvavidyalaya, Palampur
24.	**DPCaCMS-2**	Derived through back cross breeding of CMS line × DPCaf-8	CSK Himachal Pradesh Krishi Vishvavidyalaya, Palampur
25.	**DPCaCMS-3**	Derived through back cross breeding of CMS line × DPCaf-10	CSK Himachal Pradesh Krishi Vishvavidyalaya, Palampur
26.	**DPCaCMS-4**	Derived through back cross breeding of CMS line × S121Y	CSK Himachal Pradesh Krishi Vishvavidyalaya, Palampur
27.	**DPCaCMS-5**	Derived through back cross breeding of CMS line × S121W	CSK Himachal Pradesh Krishi Vishvavidyalaya, Palampur
28.	**DPCaf-CMS2**	Derived through back cross breeding of CMS line × Palam Uphar	CSK Himachal Pradesh Krishi Vishvavidyalaya, Palampur
29.	**DPCaf-CMS3**	Derived through back cross breeding of CMS line × Palam Uphar	CSK Himachal Pradesh Krishi Vishvavidyalaya, Palampur
30.	**DPCaf-CMS4**	Derived through back cross breeding of CMS line × Palam Uphar	CSK Himachal Pradesh Krishi Vishvavidyalaya, Palampur
31.	**DPCaf-CMS5**	Derived through back cross breeding of CMS line × Palam Uphar	CSK Himachal Pradesh Krishi Vishvavidyalaya, Palampur
32.	**DPCaf-CMS7**	Derived through back cross breeding of CMS line × DPCaY-7	CSK Himachal Pradesh Krishi Vishvavidyalaya, Palampur
33.	**Pusa Paushja**	-	ICAR-IARI, New Delhi
34.	**Palam Uphar (Check)**	-	CSK Himachal Pradesh Krishi Vishvavidyalaya, Palampur
35.	**Pusa Snowball K-1 (Check)**	-	ICAR-IARI, Regional Station, Katrain, Kullu, H.P.
36.	**Pusa Snowball K-25 (Check)**	-	ICAR-IARI, Regional Station, Katrain, Kullu, H.P.

### Phenotyping

The Protection of Plant Varieties and Farmers’ Rights Authority (PPV & FRA) in New Delhi, India proposed 11 agro-morphological DUS descriptors for cauliflower, along with 20 other morphological and quality traits. Keeping these morphological traits in consideration, the observations were made on randomly selected five plants of each genotype in each replication. The data was changed to the individual DUS test scores and further transformed to binary data. The data on other morphological and quality characteristics, such as days to curd initiation, days to first marketable curd harvest, stalk length (cm), leaf length (cm), leaf width (cm), number of leaves per plant, plant height (cm), plant frame (cm), curd polar diameter (cm), curd equatorial diameter (cm), curd size index (cm^2^), curd solidity (g/cm), gross plant weight (g), marketable curd weight (g), net curd weight (g), non-marketable curds (%), harvest duration (days), harvest index (%), total soluble solids (°Brix) and ascorbic acid (mg per 100g fresh weight basis) were recorded and means of all observations were calculated for further statistical analysis.

### Genotyping using SSR markers

#### DNA extraction and purification

Using the Cetyl trimethyl ammonium bromide (CTAB) procedure described by Clarke [20], DNA extraction for the genotypes was done. Using a pestle and mortar, fresh and tender leaf tissues were crushed, and the extract was then transferred to a 2 ml Eppendorf tube containing 2000 μl of 2X CTAB extraction buffer. For 40–50 minutes, tubes were incubated in a water bath at 65°C while being shaken every 10 minutes. After adding a 24:1 ratio of chloroform: isoamyl alcohol in tubes (800 μl), the mixture was shaken for 25 minutes. The samples were then centrifuged at 12000 rpm for 7 minutes. After being transferred, the supernatant (upper phase) into 1.5 ml tubes with an equivalent volume of cold isopropanol (600 μl) was chilled overnight at -20°C. The next day, centrifugation was carried out for 7 minutes at 12000 rpm. After pellet formation, the supernatant was removed and the pellets were washed in 200 μl of 70% alcohol, centrifuged at 7000 rpm for 3 minutes and then allowed to air dry. 50 μl of 1 X TAE buffer were added to the pellet to dissolve it and they were kept at -20°C. Additionally, a 1% agarose gel and the Nanodrop 2000c (Thermo Scientific) were used to verify the quantity and purity of the isolated DNA.

#### SSR primer selection

Eighty SSR markers were selected from the list of previous researchers [19, 21–23] from all nine chromosomes, reported to had steady amplification and distinct banding patterns. These 80 markers were screened on 36 genotypes. The primers which failed to produce frequent or clear band sizes i.e. monomorphic were excluded from the research. Therefore, a final set of 36 polymorphic SSR primers were selected for further investigation and scored (S1 Table).

#### PCR amplification and gel electrophoresis

The PCR reaction was conducted in a total volume of 10 μl (1 μl of template DNA and 9 μl of reaction mixture containing 0.6 μl of each forward and reverse primer, 3.5 μl of master mix, and 4.3 μl of nuclease-free water) in PCR tubes. This reaction was performed in a DNA Thermal Cycler (BIO-RAD) as follows: an initial denaturation step for 4 min at 94˚C; annealing step for 30s at 94˚C and extension step for 1 min at 72˚C followed by 35 cycles. Final extension step followed by 7 min at 72˚C and then the reaction was stored at 4 ˚C for ∞.

Each sample’s PCR product were verified by running it on a 2.5% agarose gel for two hours at 80 V with 10 μl of ethidium bromide in 1X TAE buffer. Additionally, a 100-bp DNA ladder was used as a molecular indicator for determining the size of the SSR primer product. The Gel-Documentation Unit (BIO-RAD) was used to view the gels and took pictures of them.

### Statistical analysis

#### DUS data analysis

NTSYS-pc (version 2.02) was used to create the dendrogram using binary data of 11 DUS descriptors [24]. Each descriptor’s Shannon Diversity Index (H) was determined using the following formula:

$$H=‐\sum_{i=1}^{S}p_{i}\ln p_{i}$$

where, *p*_*i*_ is the proportion (n/N)

#### Morphological diversity analysis

Each genotype’s phenotypic data for two quality and 18 morphological traits was collected, subjected to the Mahalanobis D^2^ statistic [25] and then sorted into various clusters using the Tocher technique [26]. The computer programme WINDOSTAT 8.0 created by Indostat Services was utilised to carry out the D^2^ analysis. The following equation represents the Mahalanobis D^2^ analysis between two genotypes determined based on the ’p’ characters:

$$D^{2}=\sum_{i=1}^{p}\sum_{j=1}^{p}wij(Xi1-Xi2)(Xj1-Xj2)$$

where,

wij = variance-covariance matrix

w^ij^ = reciprocal of (wij), (i j = 1,2……, p)

X_i1_ = sample mean for i^th^ character for first sample

X_i2_ = sample mean for i^th^ character for second sample

Additionally, using the EIGEN algorithm in the programme XLSTAT with the correlation coefficient between two genotypes, principal component analysis (PCA) was used to predict the grouping pattern of the 36 cauliflower genotypes.

#### Molecular analysis

The amplicons were scored as 1 (present) or 0 (absent) depending on the molecular size (base pair) of the amplified product and a dendrogram was created using Rohlf [23] NTSYS software (version 2.02) and the Jaccard’s similarity matrix coefficient.

$$J_{ij}=C_{ij}/(n_{i}+n_{j}-c_{ij})$$

where,

‘C_ij_’ is the number of positive matches between two genotypes

n_i_ and n_j_ are the total number of bands in genotype i and j, respectively, in SIMQUAL program of NTSYS-pc package (version 2.02)

After that, PCoA plot was designed using the GenALEx 6.5 software [27]. Further, binary data was used to calculate a genetic dissimilarity matrix using the Jaccard dissimilarity index (d_ij_) between pairs of accessions (units),

$$d_{ij}=(b+c)/[a+(b+c)]$$

where,

d_ij_ represents the dissimilarity between units i and j

The DARwin programme (version 6.0) was used to generate an UnWeighted Neighbour Joining tree from the dissimilarity matrix [28] and the polymorphism information content (PIC) of each gel was calculated.

$$\mathrm{PIC}=1-\sum_{i=1}^{k}P_{i}{}^{2}$$

where,

*k* is the total number of alleles

*p*_*i*_ is the frequency of the i^th^ allele in the set of genotypes investigated [29].

Various genetic diversity parameters including average number of alleles (Na) count, effective number of alleles (Ne), Shannon’s diversity index (I), observed heterozygosity (Ho), and predicted heterozygosity (He), were examined using POPGENE software version 1.32. The 36 cauliflower genotypes’ genetic makeup was determined using the Bayesian model-based programme STRUCTURE (v.2.3.3) [30]. The analysis was done with K ranging from 1 to 10 using an admixture model with 10,000 burning periods and 10,000 replicates. The final peak of plotting LnPD values were identified using online web-based Structure Harvester program. In addition to this, genetic diversity within and among the populations (AMOVA) was determined using GenAlex software ver. 6.5. [27].

## Results

### Agro-morphological characterization

**Fig 1:**
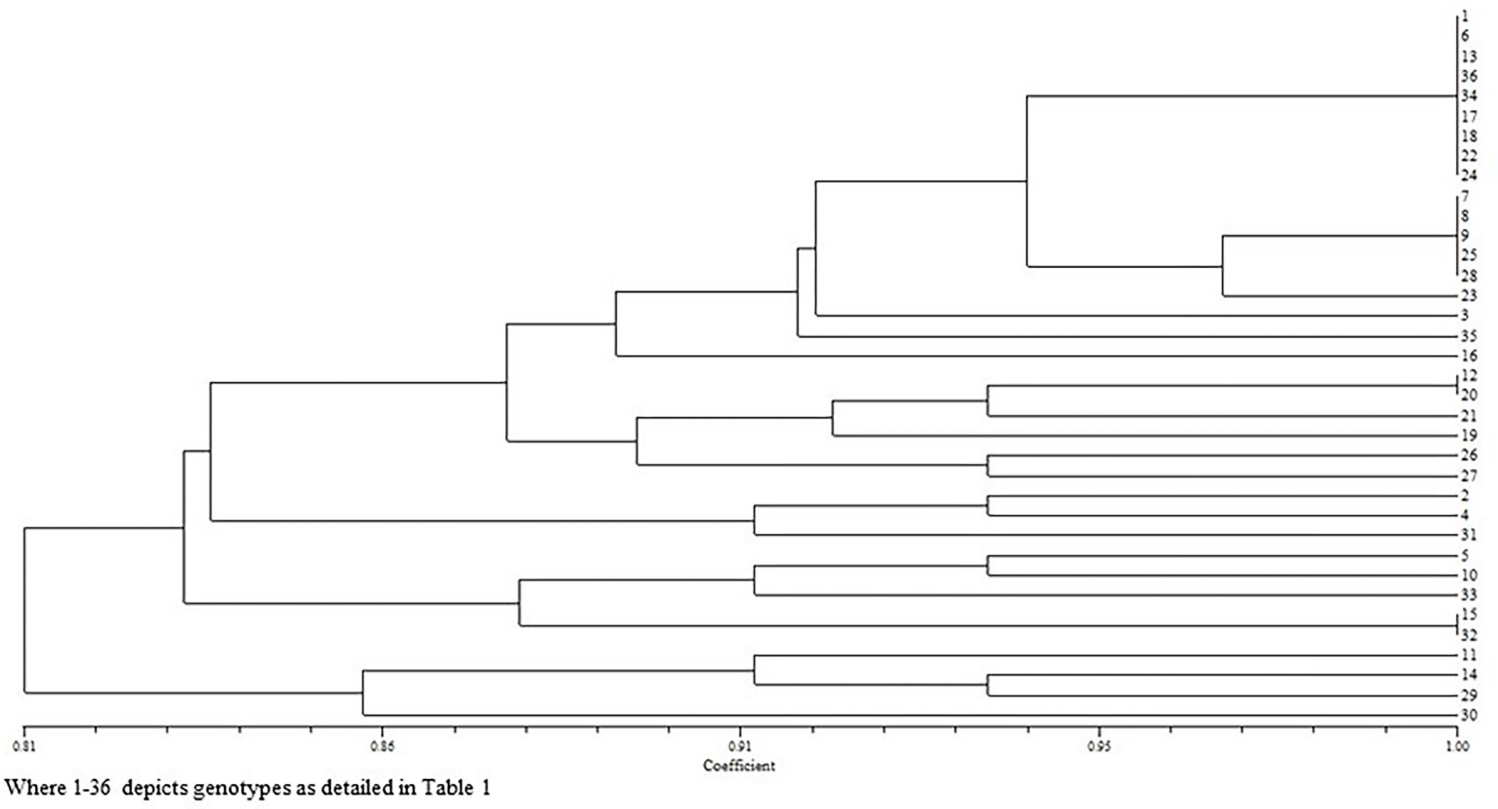
Groping of 36 genotypes based on morphological characters.

Qualitative characteristics are crucial for describing plants since they are heavily impacted by customer preferences, the socioeconomic environment and natural selection. In Table 2, the diversity index for cauliflower is shown together with the distribution (%) of several categories of minimum descriptors. The Shannon Diversity Index (H) ranged from 0.29 to 0.94 with a mean of 0.54. The similarity coefficient ranged from 0.81 to 1.00, indicating a wide range of genetic variation among the genotypes. There was significant diversity among 36 genotypes except for leaf glossiness, blanched/non-blanched curds, curd texture, curd compactness and riceyness which were determined to be monomorphic and were omitted from the research. Majority of genotypes possessed intermediate plant growth habit (72%), erect leaves (Type-3) position of leaves (78%), dark green leaf colour (75%), broad elliptic leaf shape (89%), crenate leaf margin (56%) and white curd colour (92%). The cluster analysis grouped all the genotypes into two major clusters with two sub-clusters (Fig 1 and S2 Table).

**Table 2:** Distribution (%) of different categories of minimal descriptors in cauliflower.

Trait	Scale of descriptor	Frequency	Relative frequency (%)	Shannon Diversity Index (H)
**Plant growth habit**	Dwarf	0	0.00	0.59
	Intermediate	26	72.22	
	Tall	10	27.78	
**Position of leaves**	Erect (Type-3)	28	77.78	0.53
	Semi erect (Type-2)	8	22.22	
	Horizontal	0	0.00	
**Leaf Colour**	Light green	9	25.00	0.56
	Dark green	27	75.00	
	Bluish green	0	0.00	
**Leaf Shape**	Narrow elliptic	4	11.11	0.35
	Elliptic	0	0.00	
	Broad elliptic	32	88.89	
**Leaf Margin**	Entire	12	33.33	0.94
	Crenate	20	55.56	
	Dentate	0	0.00	
	Serrate	4	11.11	
	Undulate	0	0.00	
**Curd Colour**	White	33	91.67	0.29
	Creamy white	3	8.33	
	Orange	0	0.00	0.59
**Mean**				0.54
**Maximum**				0.94
**Minimum**				0.29
**Range**				0.65

### Mahalanobis D^2^ diversity analysis

**Fig 2:**
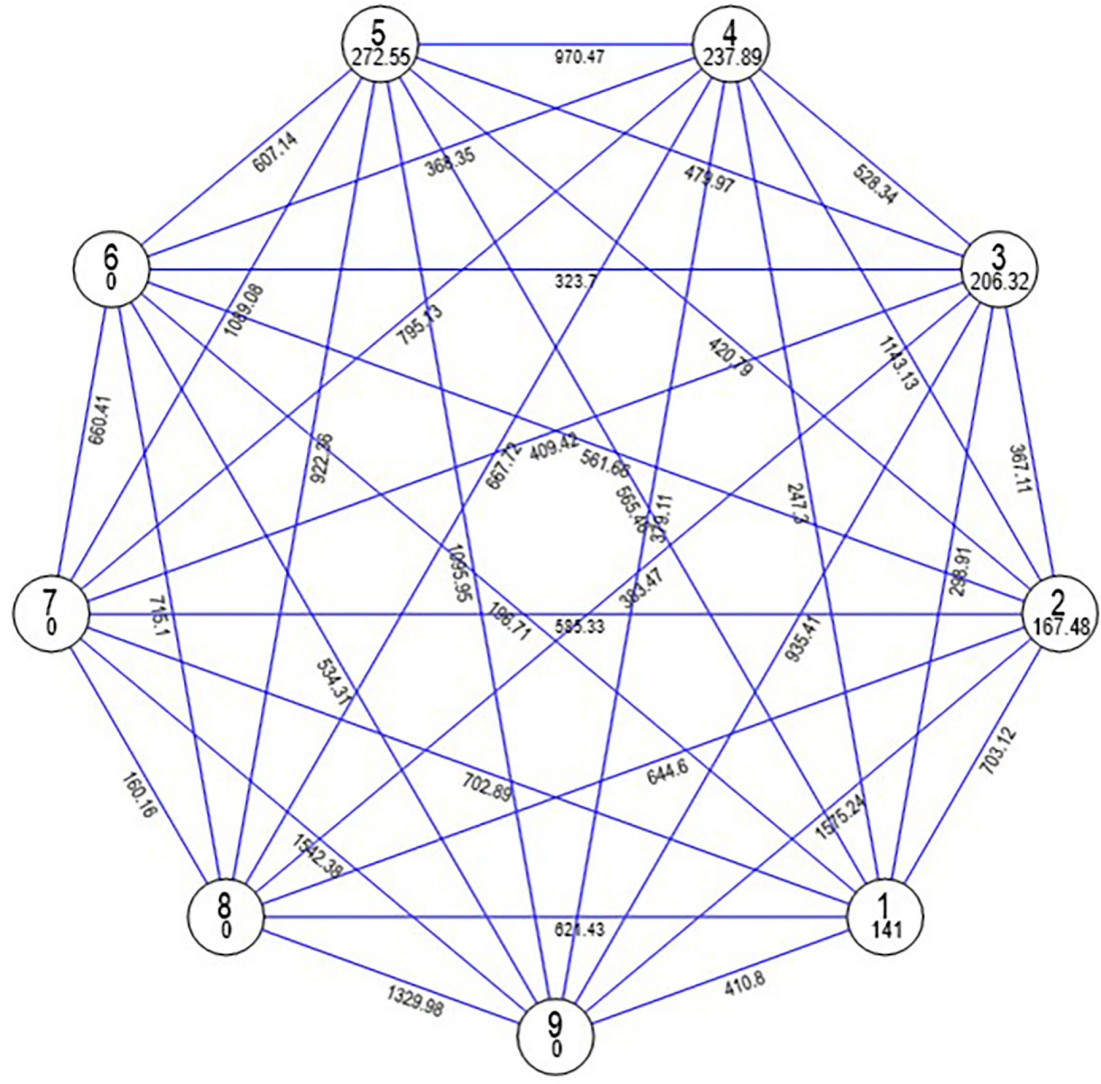
Average distance of intra and inter-cluster centroids based on various traits in cauliflower genotypes.

**Fig 3:**
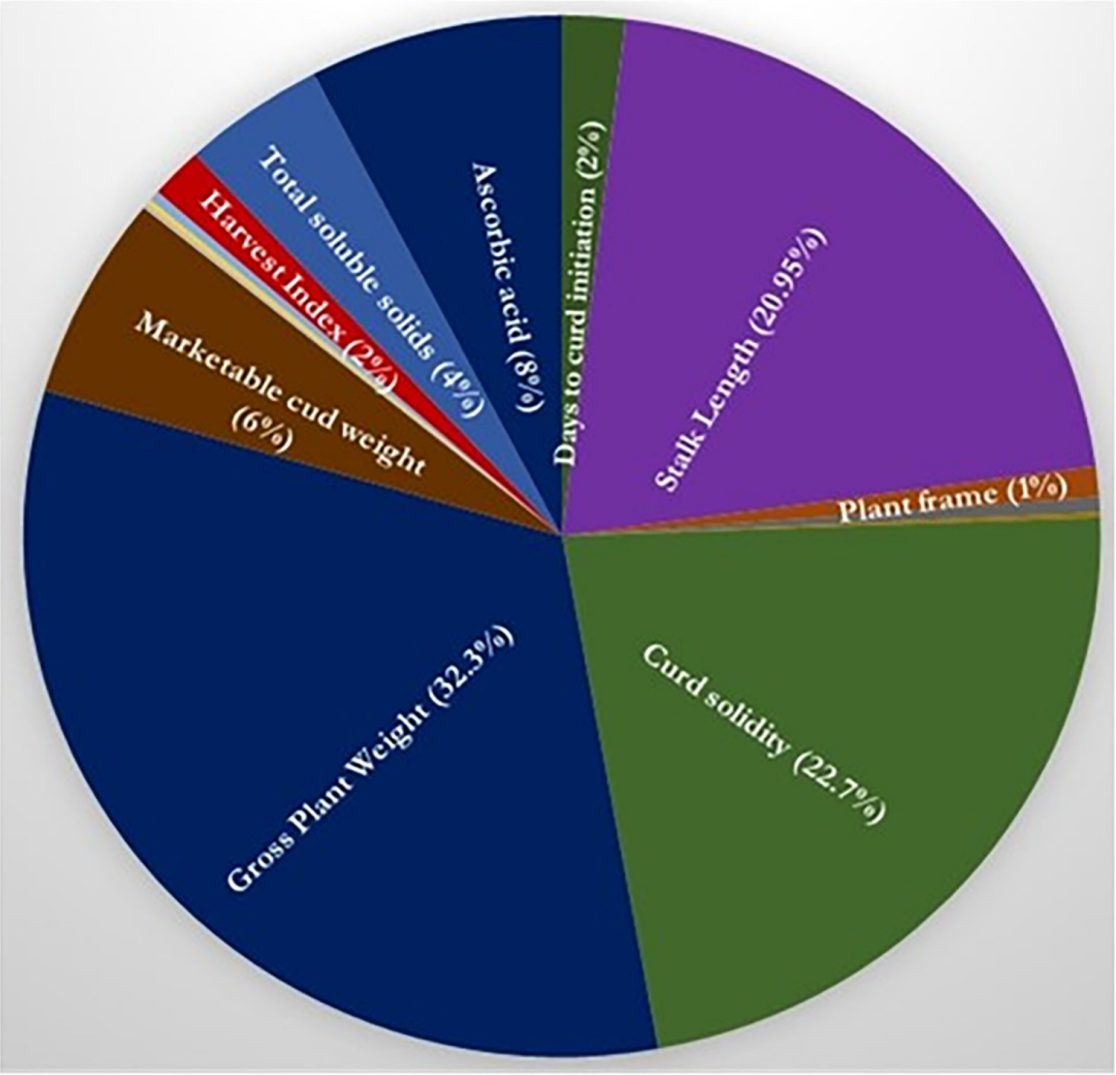
Relative contribution (%) of individual trait to the genetic divergence among cauliflower genotypes.

To differentiate the genotypes based on similarity and differences, the genotypes were subjected to multivariate cluster analysis based on Euclidean distance among the phenotypic characters. The genotypes were grouped into nine distinct cluster following Tocher’s procedure [26]. Cluster I categorized as the largest with 11 genotypes followed by 9, 5, 4 and 3 genotypes in clusters III, V, IV and II, respectively while cluster VI, VII, VIII and IX were monogenotypic that revealed optimum genetic diversity in the germplasm (Fig 2 and S3 Table). The inter-cluster distance ranged from 12.65–39.71, with maximum divergence in clusters II and IX (39.71) followed by clusters VII and IX (39.29), and clusters VIII and IX (36.50) [Table 3]. Cluster means also showed differences for all the traits (S4 Table). Cluster VII recorded maximum mean values for leaf length (37.16 cm), number of leaves per plant (12.80), plant height (46.39 cm), curd polar diameter (8.00 cm), curd size index (98.11), curd solidity (52.98), marketable curd weight (651.27 g) and net curd weight (423.87 g). Similarly, cluster VIII showed maximum means for stalk length (4.31 cm), leaf width (18.40 cm), plant frame (55.72 cm), curd equatorial diameter (12.27 cm), gross plant weight (940.60 g) and harvest duration (17 days). These mean values point towards the diversity in genotypes viz., DPCaf-CMS5 ‘DPCaCMS-1’, ‘DPCaf-29’, ‘DPCaf-US’ ‘DPCaf-W4’, ‘DPCaf-W131W’and ‘DPCaY-7’ for various important curd and plant characters namely, marketable/net curd weight, curd size index/solidity, plant frame, harvest duration. In addition, the relative contribution of individual trait indicated the maximum contribution towards genetic divergence by gross plant weight (32.38%) followed by curd solidity (22.70%) and stalk length (20.95%) [Fig 3]. Thus, the captioned parents and traits with maximum diversity can be utilized in cauliflower improvement programme.

**Table 3:** Average intra and inter-cluster values of D^2^ and √D^2^ among clusters.

Clusters	I	II	III	IV	V	VI	VII	VIII	IX
**I**	**11.88 (3.45)**	26.52 (5.15)	17.29 (4.16)	15.73 (3.97)	23.78 (4.88)	14.01 (3.74)	26.50 (5.15)	24.94 (4.99)	20.28 (4.50)
**II**		**12.94 (3.60)**	19.16 (4.38)	33.81 (5.81)	20.50 (4.53)	23.70 (4.87)	24.18 (4.92)	25.39 (5.04)	39.71 (6.30)
**III**			**14.35 (3.75)**	22.98 (4.79)	21.90 (4.68)	17.98 (4.24)	20.21 (4.50)	19.57 (4.42)	30.60 (5.53)
**IV**				**15.42 (3.93)**	31.15 (5.58)	19.19 (4.38)	28.19 (5.31)	25.85 (5.08)	19.49 (4.4)
**V**					**16.51 (4.06)**	24.61 (4.96)	32.97 (5.74)	30.36 (5.51)	33.11 (5.75)
**VI**						**0.00 (0.00)**	25.70 (5.07)	26.75 (5.17)	23.13 (4.81)
**VII**							**0.00 (0.00)**	12.65 (3.56)	39.29 (6.27)
**VIII**								**0.00 (0.00)**	36.50 (6.04)
**IX**									**0.00 (0.00)**

Bold values are intra-cluster distance

Data in parenthesis are √D^2^value

### Principal component analysis (PCA)

**Fig 4:**
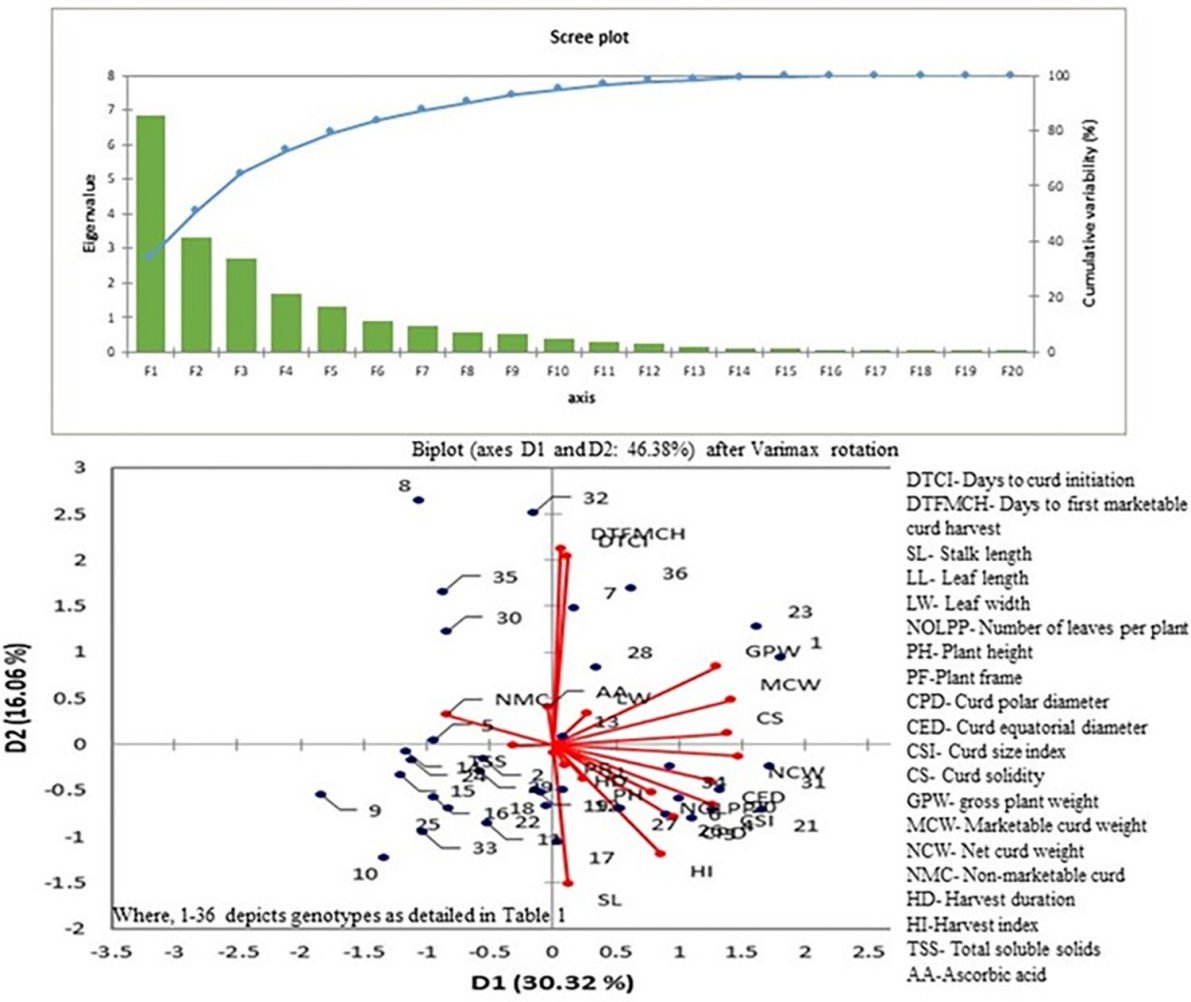
Principal component analysis (PCA) based on morphological traits for active variables (Red) and active observations (Blue).

In the present study, 36 genotypes were characterized according to 20 phenotypic traits by PCA analysis (Fig 4) that divided total variation into 20 principal components. The first six PCs were selected with eigen values of more than 1 and they cumulatively explained 79.29% of total variation. PC1 explained 27.82% of the total variability that was mainly influenced by days to curd initiation, days to first marketable curd harvest, curd polar diameter, gross plant weight and harvest index. PC2 showed the association of harvest index and harvest duration with 18.91% contribution. In PC3 (11.30%), plant frame had a major impact. The fourth main component explained 7.75% of total variability which were due to days to curd initiation, leaf width, curd size index, curd solidity and non-marketable curd (S4 Table).

### Diversity analysis using SSR markers

**Fig 5:**
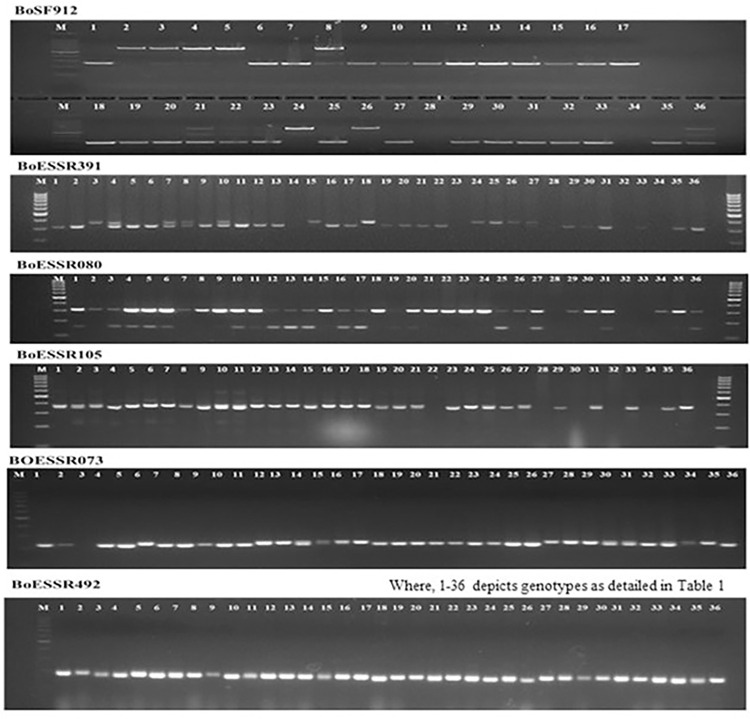
PCR amplification profile of SSR markers for 36 cauliflower genotypes (PCR product was separated on 2.5% agarose gel, M = 100bp DNA ladder).

**Fig 6:**
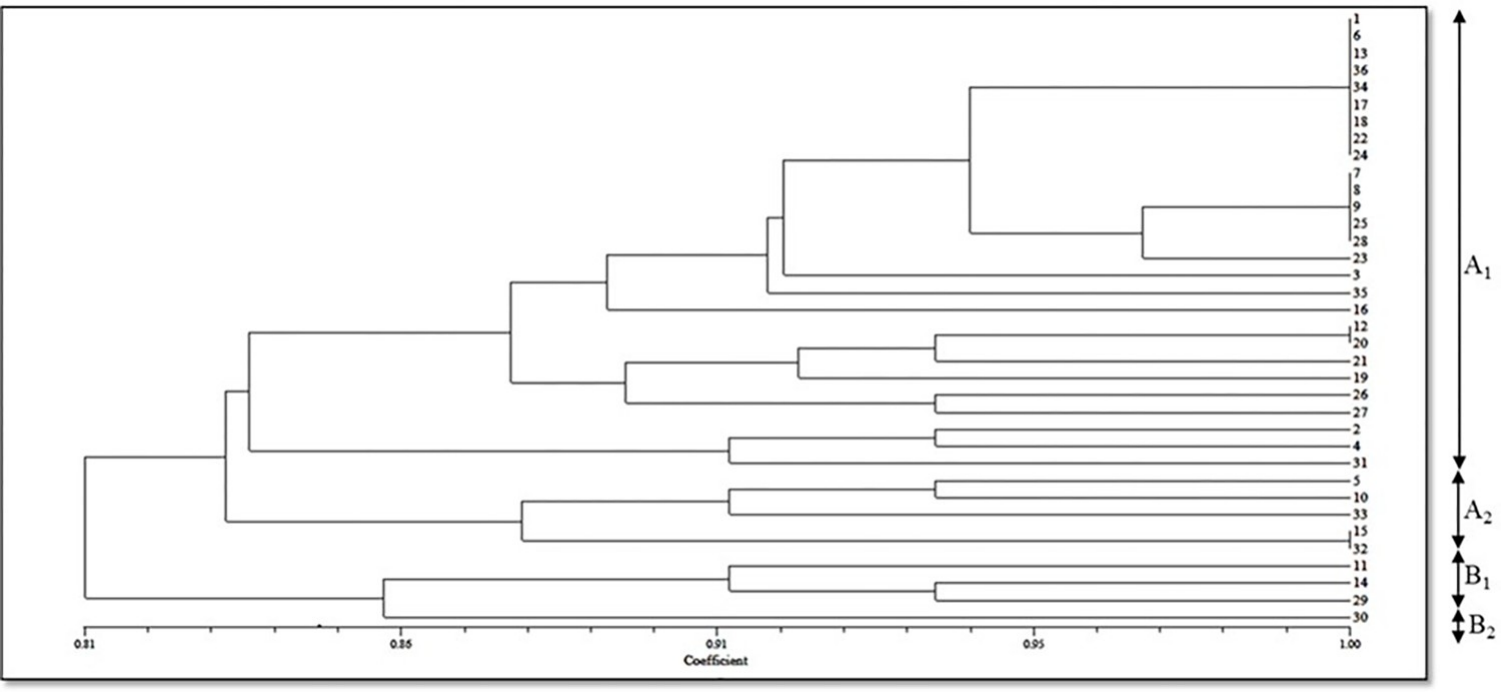
Dendrogram generated for 36 cauliflower genotypes by UPGMA (NTSYS-pc software) using SSR data.

**Fig 7:**
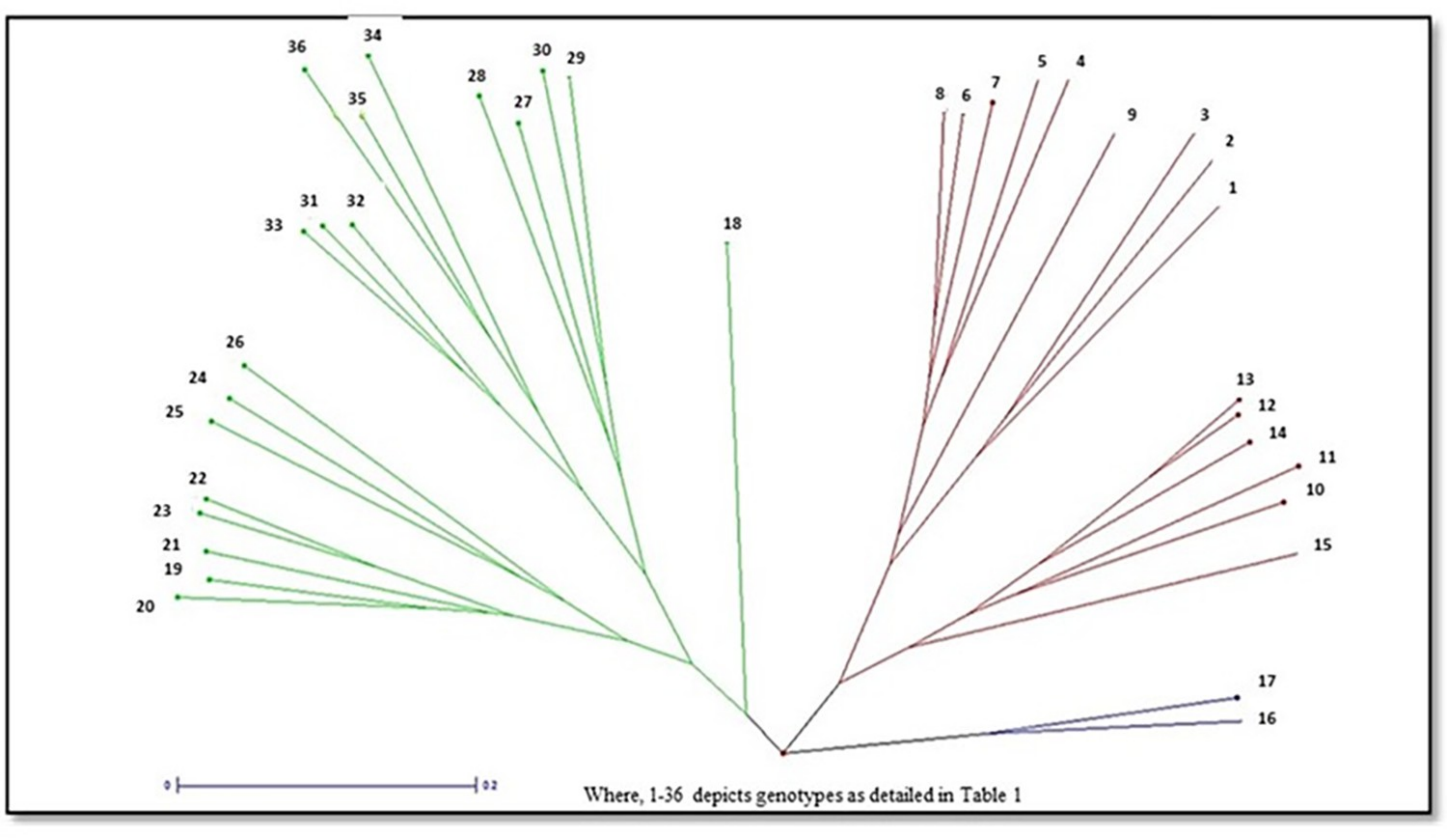
Neighbor joining tree of 36 genotypes generated by DARwin software (using data from 36 SSR markers).

**Fig 8:**
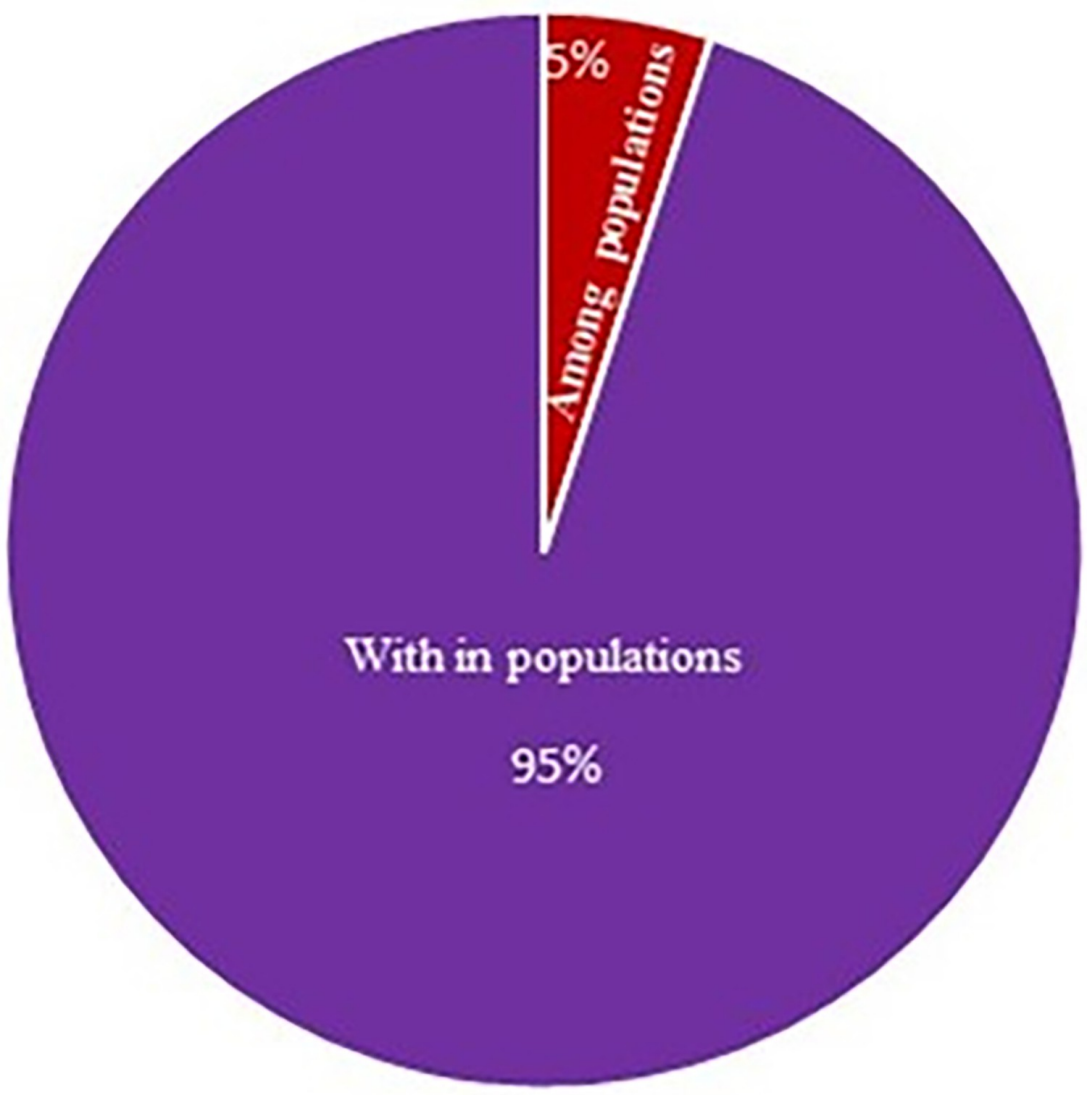
Molecular variance among and within population of cauliflower based on AMOVA.

**Fig 9:**
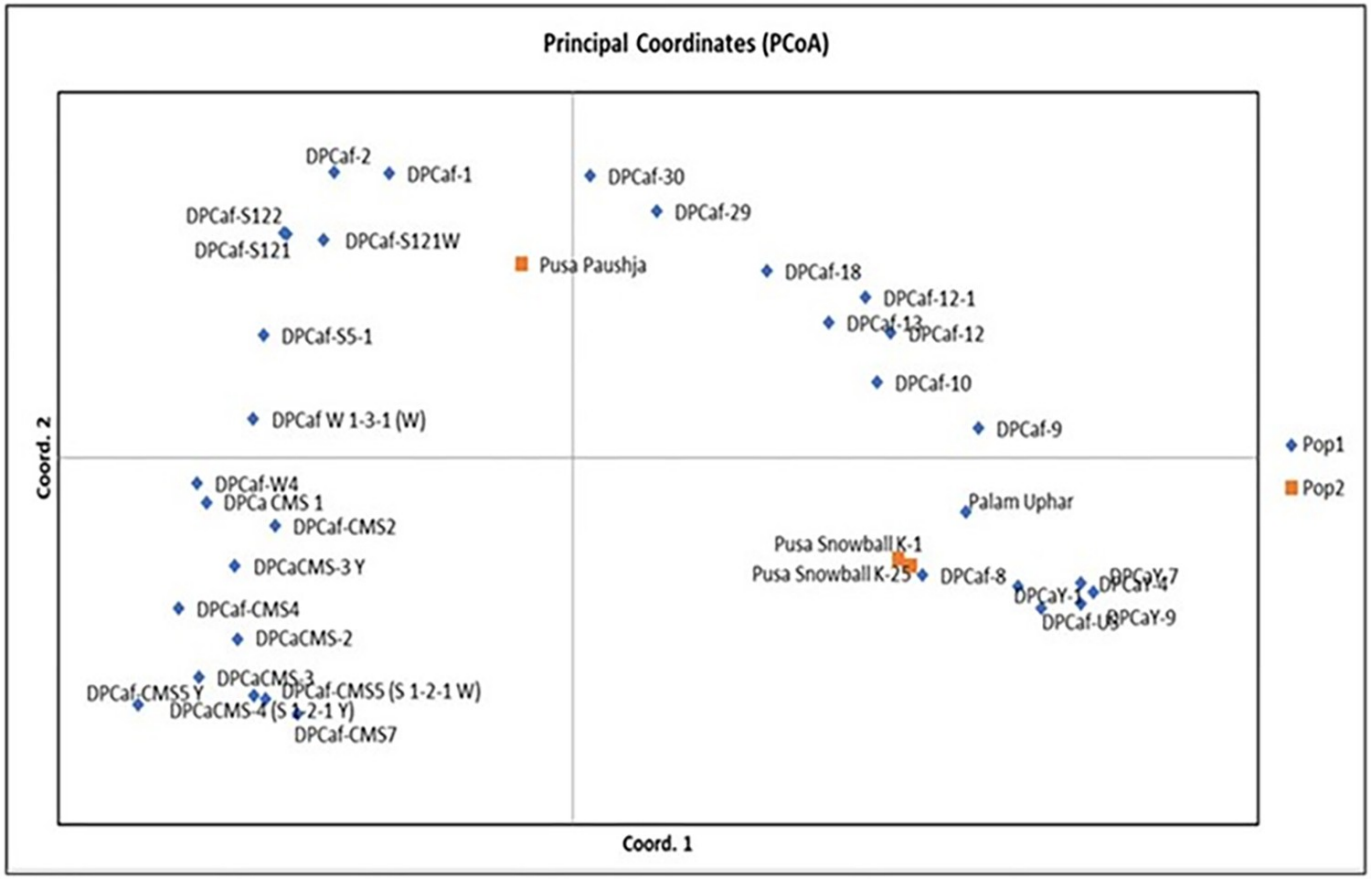
Principal coordinate analysis using SSR markers for cauliflower genotypes.

In this study, 36 SSR markers, uniformly distributed all over the nine chromosomes (S1 Table) with high polymorphism content and clear banding pattern recognition were applied for genomic DNA amplification of 36 cauliflower genotypes (Fig 5). Table 4 explains the information on different number of alleles per locus (Na), effective number of alleles per locus (Ne), Shannon’s Information index (I), polymorphism information content (PIC), observed heterozygosity and expected heterozygosity. A total of 152 alleles were observed with each marker correlating to 2–7 alleles with average of 4.22 alleles per marker. The PIC of markers differed significantly with maximum by primer BoESSR492 (0.77) and the minimum by BoESSR370 (0.11) with average value of 0.58. The UPGMA dendrogram divided 36 genotypes in two major clusters each containing 18 genotypes which further divided clusters A into two sub-clusters A1 and A2, and that of B into B1 and B2 with 8 and 10 genotypes in their respective sub-clusters (Fig 6; S5 Table). The genetic identity was further confirmed by neighbor-joining tree (Fig 7) using the DARwin software version 5.0.158 [23]. The molecular variance (AMOVA) depicts high proportion of variability within population (95%) and only 5% among population (Fig 8). Associations among the 36 genotypes following PCoA (Fig 9) revealed similar grouping of lines to that of the cluster analysis which determined 49.49% diversity mainly through three principal components (PC) (26.215% for PC1, 13.81% for PC2 and 9.53% for PC3).

**Table 4:** Details of polymorphic SSR primers used for the molecular characterization of cauliflower genotypes.

Sr. No.	Primer	Number of alleles per locus (Na)	Effective number of alleles per locus (Ne)	Shannon’s Information index (I)	Polymorphic Information Content (PIC)	Observed heterozygosity (Ho)	Expected heterozygosity (He)	Fragments size (bp)
1	BoESSR632	6	4.32	1.58	0.73	0.00	0.78	60–150
2	BoESSR726	6	3.73	1.51	0.70	0.00	0.75	90–270
3	BoESSR089	2	1.49	0.50	0.27	0.00	0.33	100–200
4	BoESSR216	6	4.66	1.64	0.75	0.00	0.80	150–240
5	BoSF063	3	2.05	0.78	0.41	0.00	0.52	280–400
6	BoESSR482	5	2.28	1.05	0.50	0.17	0.57	50–390
7	BoESSR122	4	3.26	1.26	0.64	0.00	0.70	50–350
8	BoESSR151	4	3.61	1.33	0.67	0.00	0.74	250–450
9	BoGMS0726	5	3.18	1.34	0.64	0.81	0.70	200–490
10	BoSF2615	3	2.86	1.07	0.58	0.00	0.66	100–250
11	BoESSR763	4	2.15	1.25	0.63	0.00	0.69	150–200
12	BoESSR073	5	4.26	1.50	0.73	0.00	0.78	150–380
13	BoESSR077	3	1.93	0.79	0.41	0.00	0.49	200–300
14	BoESSR766	3	2.40	0.98	0.52	0.00	0.59	150–300
15	BoESSR086	4	2.79	1.17	0.59	0.00	0.65	100–225
16	BoESSR492	6	4.87	1.68	0.77	0.00	0.81	110–250
17	BoESSR510	5	4.18	1.47	0.72	0.00	0.77	70–200
18	BoESSR186	4	1.95	0.90	0.44	0.00	0.50	300–350
19	BoESSR105	6	4.50	1.61	0.74	0.00	0.79	110–300
20	BoESSR515	3	2.24	0.94	0.49	0.00	0.56	175–225
21	BoESSR303	4	3.60	1.32	0.67	0.00	0.74	50–200
22	BoESSR333	4	3.48	1.31	0.66	0.00	0.72	200–300
23	BoSF184	4	2.67	1.16	0.58	0.00	0.64	100–200
24	BoESSR207	5	2.76	1.24	0.60	0.00	0.65	300–400
25	BoESSR863	4	3.51	1.31	0.66	0.00	0.73	200–270
26	BoSF1215	4	2.93	1.16	0.59	0.00	0.67	70–200
27	BoESSR080	6	3.69	1.48	0.69	0.52	0.74	70–300
28	BoESSR054	5	3.96	1.47	0.71	0.00	0.76	200–310
29	BoESSR403	4	2.84	1.19	0.60	0.00	0.66	150–250
30	BoESSR370	2	1.13	0.23	0.11	0.00	0.12	300–400
31	BoESSR391	4	2.53	1.08	0.55	0.00	0.62	210–380
32	BoSF912	7	4.62	1.69	0.75	0.05	0.80	230–700
33	BoESSR472	3	2.66	1.03	0.55	0.00	0.63	200–280
34	BoSF2304b	3	1.70	0.70	0.36	0.54	0.42	225–480
35	BRAS011	3	1.85	0.77	0.39	0.28	0.47	200–420
36	BoESSR763	3	2.78	1.06	0.57	0.35	0.65	200–400
	Mean	4.22	3.04	1.18	0.58	0.08	0.64	

**Fig 10:**
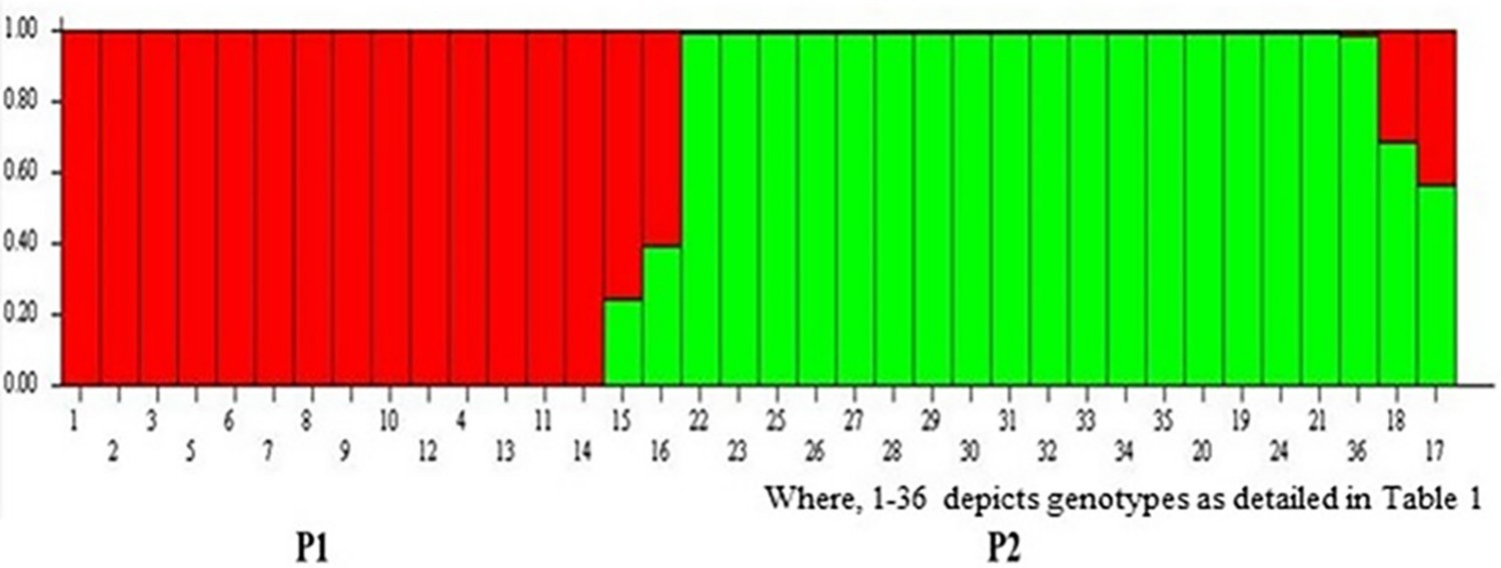
Gene pool introgression based on the population structure analysis at K = 2. Each genotype is represented by a vertical bar, which is partitioned into K colored segments that represent individual’s estimated membership coefficient (Q) to the K (= 2) clusters. Two subpopulations were P1-Red, P2-Green, Genotypes 15,16,17, and 18 showed introgression/mixed population.

Further, structure analysis revealed that the LnP(D) (log-likelihood) increased with the model parameter K value (Fig 10) with a sharp peak at K = 2, indicating that the population studied was a mixed population consisting of two subpopulations with 16 genotypes (44.44%) from CSKHPKV, Palampur in P1 and 20 genotypes in P2 (55.55%) from Palampur, IARI, New Delhi and IARI-Regional Station, Katrain, Kullu, Himachal Pradesh. Additionally, both subpopulations contained genotypes from different groups of curd maturity.

## Discussion

The first and foremost step in any efficient breeding program is to realize the extent of genetic diversity in the population of a crop species. The range of genetic diversity and population structure determine a significant role in the maintenance and augmentation of productivity [31]. The choice of the suitable genotypes would be useful based on knowledge of the genetic relationships at the molecular and plant levels. The genetic divergence in genetic material pave way to select parents with uniqueness or distinctiveness for traits of interest for utilization in hybridization to isolate superior progenies in segregating generations [32]. The Shannon Diversity Index (H) varied from 0.29 to 0.94 based on the examination of DUS data, demonstrating a broad range of genetic variation across the genotypes. Breeders are better able to understand the genetic diversity and relationships of parents by using cluster analysis and principal coordinate analysis to create suitable hybridization combinations [33]. The cluster analysis based on Euclidean distance further substantiated the diversity in our genetic material by distinguishing them based on similarities and differences. Using Tocher’s approach (D^2^ analysis) divided the genotypes into 9 diverse groups with four monogenotypic pointing towards diversity among genotypes [34, 35]. The clustering pattern showed that genotypes with the same geographical distribution fall into different clusters, demonstrating that the genetic makeup of the genotype has an impact on the clustering pattern [18]. The utilization of genotypes in hybridization from the same cluster would presumably little diverge from one another and therefore, they may not be able to synthesize desirable segregants [36, 37]. Cluster V (16.51) showed significant heterogeneity with maximum intra-cluster distance followed by cluster IV (15.42). Clusters II and IX were most divergent as depicted from maximum inter-cluster distance (39.71), suggesting to involve genotypes from these diverse clusters in hybridization to obtain transgressive segregants [38–41]. The clusters VII and VIII were found to be the most divergent based on cluster means for majority of the valuable traits (curd and plant parameters) that had direct impact on marketable curd weight [34] and can be successfully applied for the selection and identification of various parents for hybridization [38, 39, 42].

The phenotypic traits may be simplified into numerous components using principal component analysis (PCA), giving researchers the chance to choose PCs with traits that are more strongly associated with variance [35]. Only the first 6 PCs out of a total of 20 PCs were contributing more to the overall genetic variation *i*.*e*. 79.29% and cover majority of the primary traits that describe maturity, curd and plant characteristics. Similarly, Kumar et al. [43] and Singh et al. [44] also identified a comparable observation in cauliflower. Therefore, based on agro-morphological characterization, genotypes *viz*., DPCaCMS-1, DPCaf-W4 and DPCaf-US from cluster II, DPCaf-W131W, DPCaf-S121, DPCaf-S122 and DPCaf-18 from cluster III, DPCaY-9, DPCaf-13 and DPCaY-7 from cluster IV, DPCaf- 12–1 from cluster VI, DPCaf-29 from cluster VII, and DPCaf-CMS5 from cluster VIII offer greater potential as a breeding stock to be used in hybridization programmes for the isolation of transgressive segregants in cauliflower.

Environmental elements significantly affect morphological characterization since phenotype (P) is the consequence of relationship between genotype (G) and environment (E) i.e.P = G + E + GE and therefore, the worth of any diverse genetic material is not reflected at the field level. Unbiased phenotyping caused by the combination of genotype and environment can have an impact on both the grouping pattern of genotypes and the deviation of inter-/intra cluster distance [45]. These intricacies and distortions give false information about genetic divergence. The assessment of genetic diversity through DNA markers offers a rapid and accurate method for assessing genetic diversity among genotypes [32] as they are independent of environment factors [46]. The use of DNA markers supports quick screening for polymorphic loci among diverse group of genotypes. The assessment of genetic diversity in cauliflower has been undertaken by using various molecular markers including RAPD, RFLP, AFLP and SSR [1, 21]. Among these markers, SSR are locus specific, numerous, stable, widely dispersed throughout the genome, highly polymorphic due to variation in repeat units and highly informative due to their codominant nature and their usage can thus avoid the unpredictable results caused by morphological markers [46, 47]. Therefore, they are most ideal markers to study diversity in germplasm, varietal identification, marker-assisted breeding and genome mapping [46].

In present study, 36 polymorphic SSR markers generated 152 alleles with 4.22 alleles per marker along with 3.04 effective number of alleles per locus (Ne), 1.18 Shannon’s information index (I), 0.58 Polymorphic Information Content (PIC), 0.08 Observed heterozygosity (Ho) Ho and 0.64 expected heterozygosity (He) indicating good amount of diversity in 36 genotypes of cauliflower. Zhu et al. [6] observed relatively narrow genetic diversity with average PIC value (0.316) in their cauliflower inbred lines and pointed out that this may be the result of high frequency of artificial-oriented selection, inadequate polymorphism among SSR markers, and low genetic diversity in the genetic material used. However, our results with comparative high PIC value indicating optimum genetic diversity in the breeding lines which were developed through open pollination and collected from diverse sources.

The molecular markers help to differentiate breeding material by grouping them into different clusters and through principal coordinate analysis. This helps breeders to understand the genetic relationship among inbred lines and their genetic diversity which can effectively be utilized in hybridization program to obtain desirable transgressive segregants [6, 47, 48]. Accordingly, SSR markers divided genotypes into two main clusters (A and B) each of which further categorized into two sub-clusters with variable set of genotypes based on maturity group. In sub-cluster (A_2_), genotypes of mid-late group (e.g., DPCaY-7 and DPCAY-9) were placed together while certain sub- clusters had genotypes particular to late maturity group as depicted in sub-cluster B_2_ (Pusa Snowball K-1 and Pusa Snowball K-25)_._ Vanlalneihi et al. [49] and Rakshita et al. [46] also observed agro-morphological and molecular diversity in diverse set of genotypes from different maturity groups of Indian cauliflower. Cauliflower genotypes have vast ecological distribution, strong environmental adaption, capacity to survive, and evolutionary consequences all contribute to its expanded genetic diversity [50] on account of its sensitivity to temperature for curding and thereby, distribution of genotypes to different maturity groups.

The polymorphic information content (PIC) value of the primer is an important metric for determining the degree of polymorphism to be employed in molecular research. The PIC value indicated low to high levels of diversity with 8 SSR primers with moderate range (between 0.25 and 0.50), 27 SSR markers highly polymorphic (value more than 0.50) [51]. El- Eswai et al. [52] also reported similar PIC utilizing 25 genotypes from a diversified pool and 12 SSR markers in an Ireland collection. Additionally, the neighbor-joining tree using the DARwin programme added more genetic assurance by creating three significant groups. The variation in NTSYS-pc v2.02 and DARwin v6.0.21 program may be ascribed to the differentiation in clustering method i.e. former calculate similarity coefficient while other one work on dissimilarity coefficient.

The genetic differentiation based on AMOVA might be useful in breeding programs that revealed high proportion of variation (95%) within cauliflower population and 5% among populations. Yousef et al. [53] observed 6 per cent variation among population and 94 per cent variability within population. In addition, principal coordinates analysis (PCoA) provide important information about major groups as compared to cluster analysis. PCoA plotted clear separation of the genotypes collected from different locations and suggested that 49.5% of total variation cumulatively accounted by first three axes (PC1, PC2 and PC3) with their individual contribution of 26.2, 13.8 and 9.5%, respectively. Yousef et al. [53] and Stansell et al. [54] also explained the diversity using PCoA with major contribution of first three axes.

Model-based population structure analysis (STRUCTURE) depicts homogeneous mixture and provides understanding about the introgression in the present population within gene pool, thereby elucidate grouping better than dendrogram. The differentiations at K = 2 revealed almost consistency in pedigree of genotypes with introgression in few genotypes and this mix is quite obvious as breeding genotypes is a continuous process through recombination and outcrossing events [55]. Such introgression will continue to rise in the genetic resources [56]. Yousef et al. [53] showed optimum value of K = 2 while Zhu et al. [6] and Rakshita et al. [46] at K = 4 working with different sets of cauliflower germplasm.

We noticed a good range of diversity, reflected from grouping pattern of cauliflower genotypes. The outcome of the current study demonstrated a substantial genetic diversity among cauliflower genotypes based on molecular markers, their degree of polymorphism and number of detected alleles. The grouping of genotypes based on SSR markers was inconsistent with conventional grouping of the genotypes based on morphological traits and geographic origins. In cauliflower, least SSR diversity was demonstrated by Tonguc and Griffiths [57] that may be the result of selection of genotypes from a narrow gene pool. On the other hand, Astarini et al. [58], Plieske and Struss [59] and Li et al. [19] observed the effectiveness of SSR markers in assessing the diversity range in cauliflower. Hence, it would be imperative to use more markers with their distribution across nine chromosomes to undertake inclusive diversity studies in the cauliflower germplasm. The findings of this study would be utilized to characterize and identify genetic variation in cauliflower accessions for traits of preference by breeders based on farmer/consumer assessment. The diverse genotypes namely, DPCaCMS-1, DPCaf-W4, DPCaf-US, DPCaf-W131W, DPCaf-S121, DPCaf-18, DPCaf-13, DPCaf-29 and DPCaf-CMS5 can be utilized in various improvement programs of cauliflower. The use of more SSR markers will support to identify novel genes that will provide a platform in DNA fingerprinting, genome mapping and gene pyramiding.

### Conclusion

Cauliflower is a highly thermos-sensitive crop and its curd initiation and curd developmental stages are under the influence of prevailing temperatures. Therefore, for cauliflower breeders, agro-ecological diversity along with molecular characterization represents a double-edged sword. Based on morphological and molecular characterization, genotypes namely, ‘DPCaCMS-1’, ‘DPCaf-US’, ‘DPCaf-W131W’, ‘DPCaf-S121’, ‘DPCaf-W4’, ‘DPCaf-29’, ‘DPCaf-18’, ‘DPCaf-13’, ‘DPCaY-4’, ‘DPCaf-S121’, ‘DPCaf-30’ and ‘DPCaCMS-5’ were found diverse and these genotypes could be used in future hybridization programmes to either exploit heterosis or isolation of transgressive segregants with desirable horticultural traits in mid-late/late cauliflower genotypes.

## Supporting information

S1 Table List of SSR primers used for molecular studies. (DOCX)

S2 Table Clustering of cauliflower genotypes based on DUS characters. (DOCX)

S3 Table Cluster means for different traits of cauliflower genotypes based on D^2^ statistic. (DOCX)

S4 Table Principal component analysis for different traits in cauliflower genotypes. (DOCX)

S5 Table Clustering of cauliflower genotypes on the basis of SSR data (NTSYS 2.02). (DOCX)

S1 Raw image (PDF)
