# Supplementary material for: Morphological and molecular diversity in mid-late and late maturity genotypes of cauliflower
Source: PLoS One. 2023 Aug 31;18(8):e0290495. doi: 10.1371/journal.pone.0290495 (PMC10470947; doi:10.1371/journal.pone.0290495)
Supplement: S1 Table — (DOCX) [file pone.0290495.s001.docx]

**S1 Table. List of SSR primers used for molecular studies**

| **S. No.** | **Primer** | **Primers Forward Sequence** | **Primers Reverse Sequence** | **Chromosomal position** |
| --- | --- | --- | --- | --- |
| 1 | BoESSR632^*^ | CCCTGCAATTGAAAACCAGT | AAACCGTCCAAGGATCATCA | C01 |
| 2 | BoESSR726^*^ | CAATGGGTTACGCATGGTTT | CGTTTGTGAAACAGCCATTG | C01 |
| 3 | BoESSR472^*^ | AACATCGATCCGATTCTTCG | AGCTGAAGAAAAACGCCATT |  |
| 4 | BoESSR089^*^ | ATGATCAGCGAAACCACTCC | TGATACATCCCGTTTGCTCA | C01 |
| 5 | BoESSR216^*^ | GGTTTCCGCTATGTCCAGAA | CGGAAGAAGACGTTGAGGAG | C01 |
| 6 | BoSF912^*^ | CTAGATCGCCTGCAAAGAGC | AATACGGGGAGGTAACTCGG | C01 |
| 7 | BoSF063^*^ | GAATGTTTCCTCTGCTTGGC | TCAAAATCAGGAGAATCGGG | C01 |
| 8 | BoESSR482^*^ | GGAAATTCGATAAATCGAGAGC | TCCGGAGGTCCCTTTATTTT | C02 |
| 9 | BoESSR122^*^ | AATCGAAGCTATTCGCCGTA | CATGTGTTGGCTGTTTCAGG | C02 |
| 10 | BoESSR151^*^ | GCCCACTCCTATTTCCAACA | CTTGGGAAAATCACGGAAGA | C02 |
| 11 | BoSF2294a^*^ | CACCATCGTTTCTGTCCCTT | TAACCACACCTTCCGTTTCC | C02 |
| 12 | Ni4D12 | ACCACCATCCACAGAGTTCC | GCAGGACAGACTGAAAGCG |  |
| 13 | BRAS011^*^ | TGGGACGTAGTCAGTCAACAA | CCAAGTGCGAGAAGAGGAAG | C02 |
| 14 | BoGMS0726^*^ | GTTCCGAGGGTTGTTCTT | CCATCAGGTTCAGCCATAC | C02 |
| 15 | BoSF2615^*^ | CGTTGTCTCAAATCAATGGC | TCATCCATTATCATCGGGGT | C02 |
| 16 | BoESSR065 | GAGCAATGCGGCTTCTAATC | ATGGCACCAAGAGGTGAATC |  |
| 17 | BoESSR673 | GAAAGAGGGAGGGAACGAAG | ACACGGACTCGAATTGGAAC |  |
| 18 | BoESSR073^*^ | GGACTGCCAAAAGACTGAGC | ACTCGCACAGGAACCAAAAT | C03 |
| 19 | BoESSR685 | CTTTCTCCGATGCCACTCTC | GCGGAAAGAATGCAGATAGC |  |
| 20 | BoESSR077^*^ | GCTGACGAAGGAGATCAAGG | TTCTCCCTCTCCGACTTCAA |  |
| 21 | BoESSR766^*^ | CGTGTGGAAGACCCTTCACT | TTGGGGAAGTGGTAAGAACG | C03 |
| 22 | BoESSR086^*^ | ACCTCCAAACCATGACGAAG | GGTGGTGTTCTGTTGGCTTT | C03 |
| 23 | BoESSR492^*^ | GCGCAGAATCCAGATCATAG | GGCTGGAGTATGAGCGAGAC | C03 |
| 24 | BoESSR510^*^ | TCAAGTTGGAACGAGAATGTTG | ATTCTTCAATCACGCCTCCTT | C03 |
| 25 | BoESSR844 | CCGCAGTAAACTCCTCAAGC | TCATCTTGTTTAGGCACTGGA |  |
| 26 | BoESSR186^*^ | CTTCTTCTTTCGCAGCGTCT | TGAAACCATCGTCCATGAAA |  |
| 27 | BoESSR409 | GGGGATTGGTCCTAAAAAGC | ACCAAGGCATCCCTCCTTAG |  |
| 28 | BoESSR763^*^ | TCAGGCAGCTAAATTCACACA | GAATGGGCAAAAGACAGCAT | C04 |
| 29 | BoESSR105^*^ | GGAGAAGAGTCATGGCAAGG | GGAACGGCTCACTTCTCTTG | C04 |
| 30 | BoESSR108 | GAACTCCACGGAAACCGTTA | CAAGAACCAGACCGACCCTA |  |
| 31 | BoESSR087 | GTTTCCTCTTCCACCACCAA | AATCTATCAAGAGGGCCAAGG |  |
| 32 | BoESSR515^*^ | ATCAGCCCCAAACTTTCCTT | ACGTCGCTAGTCCACGTCTT |  |
| 33 | BoESSR208 | CTTGGGCACTGGATGTTCTT | CACCAAGCAAACTTGGATCA |  |
| 34 | BoESSR248 | GATGTTGGTTTTGGTTTTGG | TGCTTTTGCTTTGATGGTCT |  |
| 35 | BoESSR303^*^ | GAACCCACCTTCCTTCAACA | GCGATTTTCAGGCAGAAGTC | C04 |
| 36 | BoESSR333^*^ | CCTTGGTCTTCTCCGATGAG | ATGATCGTGAACGTCCCATT | C04 |
| 37 | BoSF184^*^ | TTGCACGTACGTCTTTGAGG | CTGCAACGAGGATGAAAACA | C04 |
| 38 | BoSF1047 | TTAAATATGTAAGCCGCCCG | TTACCAGGGATAAAAGCTGAAG |  |
| 39 | BoESSR414 | TTGATTTTGGGAGGCAAAAG | TCTGGCGGAGAAGAATCAGT |  |
| 40 | BoESSR736 | CCACGGACGGAACAATTTAT | GCTAAAAATGCACCGCTACC |  |
| 41 | BoESSR206 | GATGAAGGCTGCTCAAGCTC | CGTCCTCCTCTTCAGCAAAC |  |
| 42 | BoESSR207^*^ | TTGCTGAAGAGGAGGACGAT | CTTCATTTGCACCGGAATCT | C05 |
| 43 | BoESSR945 | CCAGCATTTACCGAATCAGAA | AACTAAAGGGGGCAAAGGAA |  |
| 44 | BoESSR343 | TTCTATGCGCCTCCCCTAAT | GTTTCTGCACATCCGAACCT |  |
| 45 | BoSF1846 | CCCTGCTTGAGATAAACCCA | CATGATGATGCCATACGCTC |  |
| 46 | BoSF2374 | AAGCGGCACTCAACATAAGC | TAACCGTTTCTTGCTTGCCT |  |
| 47 | BrSF202 | GCGATCAGATCCAAACGAAT | AAGACGATCTCTTTCGCTGC |  |
| 48 | Ol1OB11 | AAAATGTGAGGCTGTTTGGG | TTTCGCAGCAGTAAACATGG |  |
| 49 | BoSF2878 | CCTTGCGTCTGAAACATCAA | TTACCGGGAGTAAATGCAGC |  |
| 50 | BoESSR581 | AACGAAGGTGAACCATCAGG | GGCCAGGAGCTGTTACAATG |  |
| 51 | BoESSR576 | CCACGTCGGAGAAGCTAAAG | TGGGAATGATTTTTGGGAGA |  |
| 52 | BoESSR863^*^ | CCCTAAATCAACCCCAAATTCAA | CCCCCATTACCTGACTGCAAAA | C06 |
| 53 | BoESSR903 | TTCGAAAATGAGACGAGAGG | CCGTATCGGAAGAAGTGGAA |  |
| 54 | BoESSR041 | TAGCCGGAACCAAAACTGTC | TGATGCAGAAGAGCAGAGGA |  |
| 55 | BoSF1215^*^ | CACTCGTTATATTTTTCTGTCTCG | GTGAATATATCCGACCCTGTTT | C06 |
| 56 | BoE047 | GCGGCTCAGCAGAAAGAGAGAG | TGCCACAGCTGGAGAACGTAAA |  |
| 57 | BoSF250 | GCCAGAAGATCCATACTCTCATCA | GGAATGAGCAAAAACAAGAGTCC |  |
| 58 | BoSF2505 | CATCTCTCCGTTGAATCTATTTCC | ACGGTCATCCATTAGAACCGTA |  |
| 59 | BoGMS0952 | TGGATCCCAAAAGACTCAGAACA | TATTTCCCTCAGTCGAGGTCGT |  |
| 60 | BoESSR758 | ATTTCGAGGTTCTCGCCATT | AACATGTGCCCAATGATGAA |  |
| 61 | BoESSR080^*^ | GAACCGCTGAGGCAATTATG | CTTAGCGAGTCATGGGCTTC | C07 |
| 62 | BoESSR854 | TATGAGTTGGCTGTCGGTGA | TCAGTGCCTCGACAACAAAG |  |
| 63 | BoESSR523 | GGACCAAGAGTATCGCAACC | TGATCGCACAAGGAAGTGAG |  |
| 64 | BoESSR212 | AGACAAGACGCCTCGGTTTA | CGTTATGGTAGATACTCAGATGC |  |
| 65 | BoESSR053 | TTTGCCAAGAAGCCTGAAGT | TGTACCAGCTGCAACCTCTG |  |
| 66 | BoESSR370^*^ | CGGTTACAGCATCTCAGCAA | GCCTAGTCCCACCAAAATGA |  |
| 67 | BoE783 | AATGGCGGTGGTGTTGG | TTGGGCGACTAAAGAAAAAT |  |
| 68 | BoESSR054^*^ | GAAGAAGCCCATGACCTCAG | CCATTCTCACCAGGACCACT |  |
| 69 | BoESSR060 | AACAGACGGAGTGCTGAC | GTGCTTTGTTGACAGCCAGA |  |
| 70 | BoESSR403^*^ | TGAGAAGCCTGAGACCACCT | AGATCTGCGCAGCGTTTTAC | C08 |
| 71 | BoESSR074 | CGGATAAAGGGCACATGAGT | TTTTGAATCTCAGCGACCAA |  |
| 72 | BoESSR553 | ATACAGGCGCTTTTCATTGG | AACAGACCAAGCGTTCTCTC |  |
| 73 | BoESSR934 | GTTCGCCAAATCCAAAAGC | ATCTCACTTTCGCCATTGCT |  |
| 74 | BoESSR935 | GGCTTCTCGCTAATTTCATT | CGCCTCGATCAATCTTCTT |  |
| 75 | BoESSR391^*^ | GCGACCTGTTGAAGAAGGAG | TTCTCCGCAAGAAATACAAGG |  |
| 76 | BoESSR453 | ACACGTGGATTGTGATCGAA | CTAGCGAAACCCAAAACGAG |  |
| 77 | BoESSR901 | AGCTAGCAGCGAAGTTCGAG | TTTGTCGGTGGAGAGGATTC |  |
| 78 | BoESSR920 | CATGTACCGGAACTTATTGG | GGAGGAGGGTCCTCTCAATC |  |
| 79 | BoESSR035 | TGAGGTATCACACCGATCCA | CGTTGAAGAATTGCGATTTG |  |
| 80 | BoSF2304b^*^ | AGAAACGCAAATGGTCTTCG | CTGTATGAGCATGGCTTCCA | C09 |

(*) represents polymorphic markers
