# Supplementary material for: Morphological and molecular diversity in mid-late and late maturity genotypes of cauliflower
Source: PLoS One. 2023 Aug 31;18(8):e0290495. doi: 10.1371/journal.pone.0290495 (PMC10470947; doi:10.1371/journal.pone.0290495)
Supplement: S2 Table — (DOCX) [file pone.0290495.s002.docx]

**S2 Table. Clustering of cauliflower genotypes based on DUS characters**

| **Cluster No.** | **Sub-cluster** | **No. of genotypes in each cluster** | **Genotypes** |
| --- | --- | --- | --- |
| **A** | A_1_ | 27 | DPCaf-US, DPCaf-W131W, DPCaf-2, Pusa Snowball K-25, Palam Uphar, DPCaf-12, DPCaf-12-1, DPCaf- 30, DPCaCMS-2, DPCaf-W4, DPCaY-1, DPCaY-4, DPCaCMS-3, DPCaf-CMS2, DPCaCMS-1, DPCaf-S121, Pusa Snowball K-1, DPCaf-10, DPCaf-1, DPCaf-18, DPCaf-29, DPCaf-13, DPCaCMS-4, DPCaf-CMS5, DPCaf-S121W, DPCaf-S122, DPCaf-CMS5 |
|  | A_2_ | 5 | DPCaf-S5-1, DPCaY-7, Pusa Paushja, DPCaf-9, DPCaf-CMS7 |
| **B** | B_1_ | 3 | DPCaY-9, DPCaf-8, DPCaf-CMS3 |
|  | B_2_ | 1 | DPCaf-CMS4 |
