# Supplementary material for: Morphological and molecular diversity in mid-late and late maturity genotypes of cauliflower
Source: PLoS One. 2023 Aug 31;18(8):e0290495. doi: 10.1371/journal.pone.0290495 (PMC10470947; doi:10.1371/journal.pone.0290495)
Supplement: S3 Table — (DOCX) [file pone.0290495.s003.docx]

**S3 Table. Cluster means for different traits of cauliflower genotypes based on D^2^ statistic**

| **Clusters** | | | | | | | | | | | | |
| --- | --- | --- | --- | --- | --- | --- | --- | --- | --- | --- | --- | --- |
| **Traits** | **I** | **II** | **III** | **IV** | **V** | **VI** | **VII** | **VIII** | **IX** | **Mean** | **Maximum** | **Minimum** |
| **Days to curd initiation** | 71.67 | 91.00 | 71.85 | 65.50 | 100.53 | 66.33 | 75.67 | 86.33 | 71.33 | 77.80 | 100.53 | 65.5 |
| **Days to first marketable curd harvest** | 93.76 | 106.67 | 94.56 | 91.50 | 114.80 | 92.33 | 92.00 | 99.33 | 91.67 | 97.40 | 114.8 | 91.5 |
| **Stalk length** | 2.83 | 1.94 | 2.80 | 3.52 | 2.00 | 2.45 | 3.80 | 4.31 | 2.77 | 2.94 | 4.31 | 1.94 |
| **Leaf length** | 31.30 | 29.55 | 32.18 | 32.01 | 30.99 | 29.70 | 37.16 | 35.96 | 28.94 | 31.98 | 37.16 | 28.94 |
| **Leaf width** | 15.14 | 15.29 | 15.75 | 16.53 | 16.04 | 14.63 | 14.60 | 18.40 | 15.12 | 15.72 | 18.4 | 14.6 |
| **Number of leaves per plant** | 11.29 | 11.00 | 11.49 | 10.87 | 11.19 | 12.07 | 12.80 | 12.07 | 11.47 | 11.58 | 12.8 | 10.87 |
| **Plant height** | 38.08 | 36.97 | 39.43 | 41.33 | 37.78 | 33.73 | 46.39 | 43.53 | 33.85 | 39.01 | 46.39 | 33.73 |
| **Plant frame** | 48.87 | 47.55 | 50.41 | 54.53 | 47.65 | 37.97 | 50.40 | 55.72 | 44.01 | 48.57 | 55.72 | 37.97 |
| **Curd polar diameter** | 7.02 | 7.34 | 7.47 | 7.06 | 6.72 | 7.45 | 8.00 | 7.95 | 6.54 | 7.28 | 8 | 6.54 |
| **Curd equatorial diameter** | 10.10 | 11.32 | 11.22 | 10.43 | 10.19 | 10.97 | 12.26 | 12.27 | 9.89 | 10.96 | 12.27 | 9.89 |
| **Curd size index** | 71.09 | 83.24 | 84.10 | 73.60 | 68.29 | 81.71 | 98.11 | 97.44 | 64.68 | 80.25 | 98.11 | 64.68 |
| **Curd solidity** | 35.07 | 50.92 | 45.53 | 33.59 | 37.85 | 33.70 | 52.98 | 50.85 | 20.75 | 40.14 | 52.98 | 20.75 |
| **Gross plant weight** | 632.33 | 917.09 | 816.07 | 602.10 | 820.84 | 646.67 | 933.07 | 940.60 | 428.07 | 748.54 | 940.6 | 428.07 |
| **Marketable curd weight** | 400.48 | 604.57 | 514.26 | 402.62 | 478.55 | 474.07 | 651.27 | 608.93 | 285.36 | 491.12 | 651.27 | 285.36 |
| **Net curd weight** | 246.00 | 374.80 | 340.86 | 234.87 | 252.93 | 250.93 | 423.87 | 404.00 | 135.53 | 295.98 | 423.87 | 135.53 |
| **Non marketable curd** | 17.04 | 13.68 | 15.18 | 20.72 | 22.39 | 12.04 | 10.83 | 13.33 | 28.98 | 17.13 | 28.98 | 10.83 |
| **Harvest duration** | 14.12 | 13.67 | 14.33 | 13.92 | 12.87 | 12.67 | 7.33 | 16.67 | 9.00 | 12.73 | 16.67 | 7.33 |
| **Harvest Index** | 63.65 | 66.07 | 63.01 | 66.31 | 58.46 | 73.31 | 69.79 | 64.74 | 66.61 | 65.77 | 73.31 | 58.46 |
| **Total soluble solids** | 7.76 | 6.76 | 7.51 | 7.78 | 8.44 | 6.70 | 7.29 | 6.52 | 8.31 | 7.45 | 8.44 | 6.52 |
| **Ascorbic acid** | 13.28 | 14.15 | 12.83 | 13.97 | 17.10 | 9.87 | 10.80 | 16.06 | 20.31 | 14.18 | 20.31 | 9.87 |
