# Supplementary material for: Morphological and molecular diversity in mid-late and late maturity genotypes of cauliflower
Source: PLoS One. 2023 Aug 31;18(8):e0290495. doi: 10.1371/journal.pone.0290495 (PMC10470947; doi:10.1371/journal.pone.0290495)
Supplement: S4 Table — (DOCX) [file pone.0290495.s004.docx]

**S4 Table. Principal component analysis for different traits in cauliflower genotypes**

| **Canonical Root Analysis (P. C. A.)** | | | | | | |
| --- | --- | --- | --- | --- | --- | --- |
|  | **1** | **2** | **3** | **4** | **5** | **6** |
| **Days to curd initiation** | 0.32 | 0.06 | 0.02 | 0.21 | 0.26 | 0.01 |
| **Days to first marketable curd harvest** | **0.33** | 0.12 | 0.04 | 0.06 | 0.29 | -0.19 |
| **Stalk length** | -0.36 | -0.14 | 0.11 | 0.04 | 0.21 | 0.08 |
| **Leaf length** | 0.23 | -0.09 | 0.43 | 0.06 | 0.10 | -0.16 |
| **Leaf width** | -0.11 | 0.04 | -0.21 | 0.12 | **0.61** | -0.03 |
| **Number of leaves per plant** | 0.19 | -0.17 | -0.17 | -0.40 | -0.08 | -0.16 |
| **Plant height** | -0.05 | -0.17 | 0.41 | -0.08 | -0.23 | 0.37 |
| **Plant frame** | -0.19 | -0.13 | **0.46** | 0.01 | 0.24 | 0.04 |
| **Curd polar diameter** | 0.24 | -0.22 | -0.15 | 0.01 | -0.15 | 0.09 |
| **Curd equatorial diameter** | 0.20 | -0.33 | -0.24 | -0.17 | -0.04 | 0.02 |
| **Curd size index** | -0.32 | -0.18 | -0.05 | 0.14 | 0.17 | 0.12 |
| **Curd solidity** | 0.06 | -0.43 | -0.11 | 0.19 | 0.11 | 0.28 |
| **Gross plant weight** | 0.32 | -0.25 | 0.00 | 0.07 | 0.21 | 0.13 |
| **Marketable curd weight** | -0.28 | 0.01 | -0.21 | 0.05 | -0.01 | -0.41 |
| **Net curd weight** | -0.19 | -0.29 | -0.24 | -0.26 | -0.02 | 0.18 |
| **Non marketable curd** | 0.06 | **0.28** | -0.15 | 0.13 | 0.05 | **0.61** |
| **Harvest duration** | -0.05 | 0.22 | -0.29 | **0.48** | -0.24 | 0.10 |
| **Harvest Index** | 0.25 | 0.27 | 0.12 | -0.05 | -0.07 | 0.14 |
| **Total soluble solids** | -0.17 | 0.34 | 0.09 | -0.36 | 0.03 | 0.14 |
| **Ascorbic acid content** | 0.06 | 0.21 | -0.17 | -0.48 | 0.35 | 0.20 |
| **Eigen Value (Root)** | **5.56** | **3.78** | **2.26** | **1.55** | **1.44** | **1.26** |
| **% Var. Exp.** | **27.82** | **18.91** | **11.30** | **7.75** | **7.20** | **6.31** |
| **Cumulative Var. Exp.** | **27.82** | **46.73** | **58.03** | **65.78** | **72.98** | **79.29** |
