# Supplementary material for: Morphological and molecular diversity in mid-late and late maturity genotypes of cauliflower
Source: PLoS One. 2023 Aug 31;18(8):e0290495. doi: 10.1371/journal.pone.0290495 (PMC10470947; doi:10.1371/journal.pone.0290495)
Supplement: S5 Table — (DOCX) [file pone.0290495.s005.docx]

**S5 Table. Clustering of cauliflower genotypes on the basis of SSR data (NTSYS 2.02)**

| **Cluster No.** | **Sub-cluster** | **No. of genotypes in each cluster** | **Genotypes** |
| --- | --- | --- | --- |
| **A** | A_1_ | 8 | DPCaf-US, DPCaf-S121W, DPCaf-S121, ‘DPCaf-S122, DPCaf-S5-1, DPCaf-W131W, DPCaY-1, DPCaf-W4 |
|  | A_2_ | 10 | DPCaY-4, DPCaY-7, DPCaY-9, DPCaf-1, DPCaf-2, DPCaf-8, DPCaf-9, DPCaf-10, DPCaf-12, DPCaf-12-1 |
| **B** | B_1_ | 8 | DPCaf-13, DPCaf-18, DPCaf-3’, DPCaCMS-1, DPCaf-29, DPCaCMS-2, DPCaCMS-4, DPCaCMS-3 |
|  | B_2_ | 10 | DPCaCMS-5, DPCaf-CMS2, DPCaf-CMS3, DPCaf-CMS4, DPCaf-CMS5, DPCaf-CMS7, Pusa Paushja, Palam Uphar, Pusa Snowball K-1, Pusa Snowball K-25 |
