# Supplementary material for: Morphological and molecular diversity in mid-late and late maturity genotypes of cauliflower
Source: PLoS One. 2023 Aug 31;18(8):e0290495. doi: 10.1371/journal.pone.0290495 (PMC10470947; doi:10.1371/journal.pone.0290495)
Supplement: S1 Raw image — (PDF) [file pone.0290495.s006.pdf]

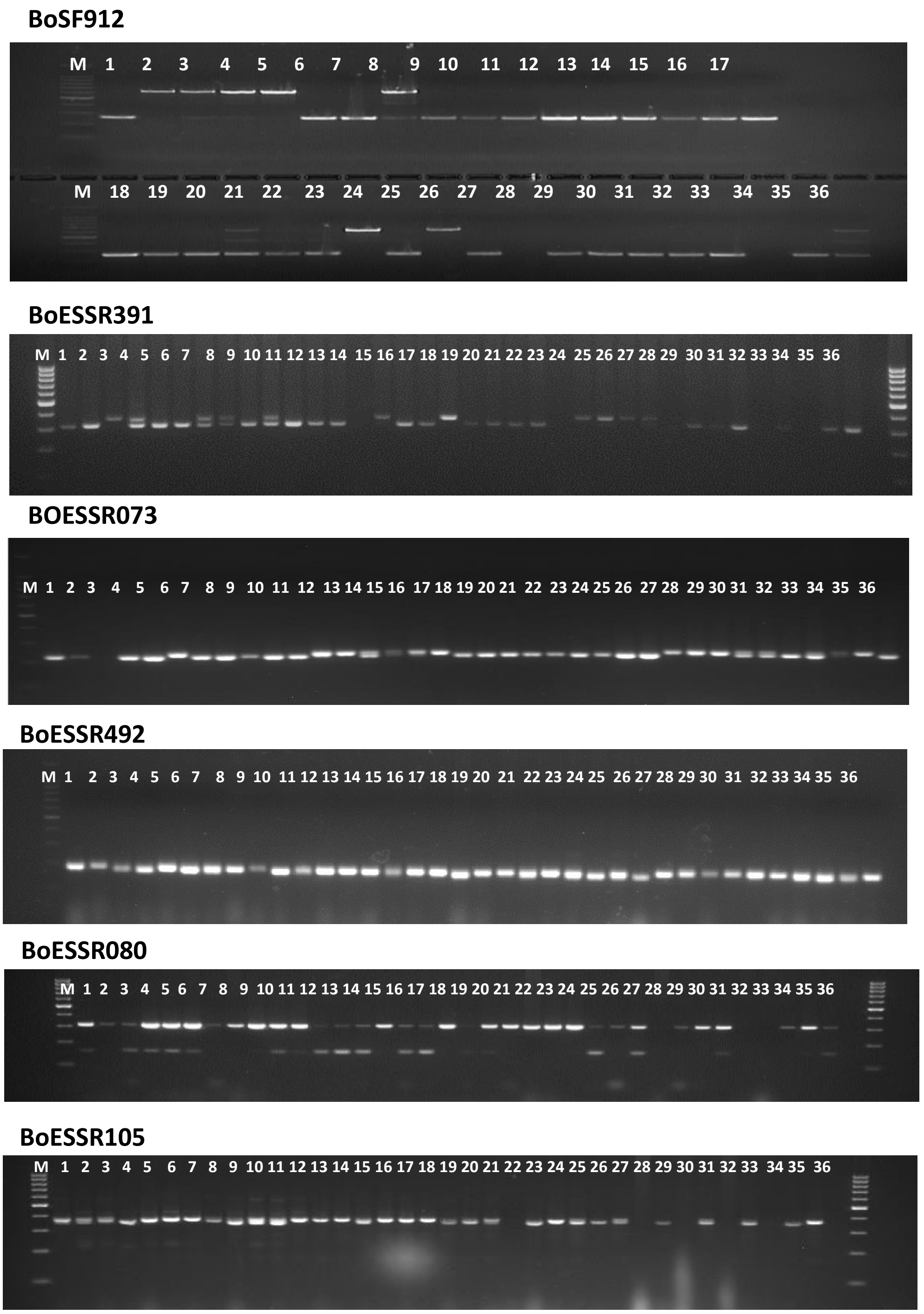

Fig 5. PCR amplification profile of SSR markers for 36 cauliflower genotypes (PCR product was separated on 2.5% agarose gel, M=100bp DNA ladder)
